# The Connexin 43 Regulator Rotigaptide Reduces Cytokine-Induced Cell Death in Human Islets

**DOI:** 10.3390/ijms21124311

**Published:** 2020-06-17

**Authors:** Seyed Mojtaba Ghiasi, Jakob Bondo Hansen, Dan Ploug Christensen, Björn Tyrberg, Thomas Mandrup-Poulsen

**Affiliations:** 1 Section of Cell Biology and Functional Genomics, Division of Diabetes, Endocrinology and Metabolism, Department of Metabolism, Digestion and Reproduction, Faculty of Medicine, Imperial College London, London W12 0NN, UK; s.ghiasi@imperial.ac.uk; 2 Department of Biomedical Sciences, University of Copenhagen, 2200 Copenhagen N, Denmark; 3 Novo Nordisk Foundation Center for Basic Metabolic Research, University of Copenhagen, 2200 Copenhagen N, Denmark; jbh@embarkbiotech.com; 4 Department of Biology, University of Copenhagen, 2200 Copenhagen N, Denmark; znf308@alumni.ku.dk; 5 Department of Physiology, Sahlgrenska Academy, Gothenburg University, 40530 Gothenburg, Sweden; bjorn.tyrberg@servier.com; 6 Cardiovascular and Metabolic Diseases, Institute de recherche Servier, 92150 Suresnes, France

**Keywords:** beta cell, inflammation, insulin, NF-κB, gap junctions

## Abstract

Background: Intercellular communication mediated by cationic fluxes through the Connexin family of gap junctions regulates glucose-stimulated insulin secretion and beta cell defense against inflammatory stress. Rotigaptide (RG, ZP123) is a peptide analog that increases intercellular conductance in cardiac muscle cells by the prevention of dephosphorylation and thereby uncoupling of Connexin-43 (Cx43), possibly via action on unidentified protein phosphatases. For this reason, it is being studied in human arrhythmias. It is unknown if RG protects islet cell function and viability against inflammatory or metabolic stress, a question of considerable translational interest for the treatment of diabetes. Methods: Apoptosis was measured in human islets shown to express Cx43, treated with RG or the control peptide ZP119 and exposed to glucolipotoxicity or IL-1β + IFNɣ. INS-1 cells shown to lack Cx43 were used to examine if RG protected human islet cells via Cx43 coupling. To study the mechanisms of action of Cx43-independent effects of RG, NO, IkBα degradation, mitochondrial activity, ROS, and insulin mRNA levels were determined. Results: RG reduced cytokine-induced apoptosis ~40% in human islets. In Cx43-deficient INS-1 cells, this protective effect was markedly blunted as expected, but unexpectedly, RG still modestly reduced apoptosis, and improved mitochondrial function, insulin-2 gene levels, and accumulated insulin release. RG reduced NO production in Cx43-deficient INS-1 cells associated with reduced iNOS expression, suggesting that RG blunts cytokine-induced NF-κB signaling in insulin-producing cells in a Cx43-independent manner. Conclusion: RG reduces cytokine-induced cell death in human islets. The protective action in Cx43-deficient INS-1 cells suggests a novel inhibitory mechanism of action of RG on NF-κB signaling.

## 1. Introduction

By inducing beta cell endoplasmic reticulum stress and apoptosis via the intrinsic mitochondrial pathway, proinflammatory cytokines have been implicated as mediators of beta cell failure and destruction causing type 1 diabetes (T1D) and type 2 diabetes (T2D) [1]. Antagonism of the action of the prototypic proinflammatory cytokine interleukin-1 (IL-1) improves beta cell function in T2D patients [2], an effect sustained 39 weeks beyond cessation of the antagonism in responders [3]. Despite a strong preclinical rationale [4], anti-IL-1 monotherapy was ineffective overall in recent-onset T1D. However, IL-1 antagonism did moderate inflammation and caused a 2.5-fold higher secretory function in T1D patients with intermediary beta cell function at baseline [5,6]. Since IL-1-induced beta cell apoptosis is potentiated by other proinflammatory cytokines, such as TNFα, IFNɣ, and IL-6, and since anti-TNF therapy improved beta cell function in a small placebo-controlled trial [7], it is likely that a combination of treatments targeting various aspects of signaling caused by the cytokine network is needed to improve the efficacy of anti-cytokine strategies in T1D, as has indeed been demonstrated in animal models [8]. Thus, there is a need for novel safe therapeutic approaches for such combination therapies.

Appropriate pulsatile insulin secretion depends on islet intercellular communication and synchronization [9]. Accordingly, cytokine-mediated de-synchronization of intercellular oscillating calcium fluxes alters the beta cell transcriptome and sensitizes it to a stress-induced impaired secretory function and apoptosis [10]. Gap junctions are intercellular channels composed of two connexin (Cx) hemi-channels in homo- or heterotypic combinations, with each hemi-channel comprising of six Cx subunits [11]. Cx36 is a predominant Cx in beta cells and regulates insulin secretion by calcium flux synchronization [11,12,13]. Other Cxs are also expressed in islets (Cx43 and Cx45 in mice; Cx30.3, Cx31, Cx31.1, Cx31.9, Cx43, and Cx45 in humans), although the function of these Cxs is less characterized [11,14,15,16]. Cx43 was recently identified as an important regulator of beta cell differentiation [17] and maturation [18], explaining why beta cell-specific knockout of Cx43, but not of Cx36, reduces the pancreatic insulin content and islet size [19]. However, Cx43 was not found to be expressed on adult beta cells or insulin-producing cell lines [20,21,22]. Interestingly, Cx43 and Cx32 are expressed on non-endocrine pancreatic cells, and heterotypic channels between, e.g., endothelial cells and islet endocrine cells [23,24] have been implicated in regulating glucose-stimulated islet blood flow [25]. Whole-body Cx36-deficient mice develop beta cell destruction and hyperglycemia, whereas beta cell-specific transgenic Cx36 overexpression protects against single high-dose streptozotocin-induced diabetes and restores islet insulin contents in this model. In addition, proinflammatory cytokines reduce beta cell Cx36 expression, and Cx36 deficiency aggravates cytokine-induced beta-cell toxicity [10]. Therefore, pharmacological targeting of gap junctions has been proposed as a novel approach to rescue and restore the pancreatic beta cell mass from stress-induced apoptosis. An important knowledge gap to guide clinical trials is the demonstration of the importance of the Cx family in human islet apoptosis induced by inflammatory stress.

The peptide rotigaptide (RG) increases intercellular conductance in cardiac muscle cells and restores gap junction intercellular communication (GJIC) in atrial cardiomyocytes of metabolically stressed rats [26]. RG prevents dephosphorylation and thereby uncoupling of Cx43, possibly by acting on yet unidentified protein phosphatases [27,28]. In addition, RG dose-dependently increases the Cx43 expression level in rat cardiomyocytes [29]. Interestingly, proinflammatory cytokines downregulate Cx43 expression and inhibit GJIC in human corneal fibroblasts [30] and in mouse astrocytes [31], suggesting that Cx43 plays a critical role in regulating the GJIC in inflammatory stress in cells. However, the role of Cx43 in the response of pancreatic islet cells to cytokine stress is yet unclear. Here, we had two aims: (1) To investigate if Cx43 plays a role in inflammatory stress-induced human islet cell apoptosis using RG as a Cx43 coupler, and (2) to examine if RG exerts an anti-apoptotic effect via Cx43 in human islets by demonstrating loss-of-function of RG on cytokine-induced apoptosis in Cx43-deficient INS-1 cells.

## 2. Results

### 2.1. Rotigaptide Reduces Cytokine-Induced Apoptosis in Cx43-Expressing Human Islets

**Figure 1:**
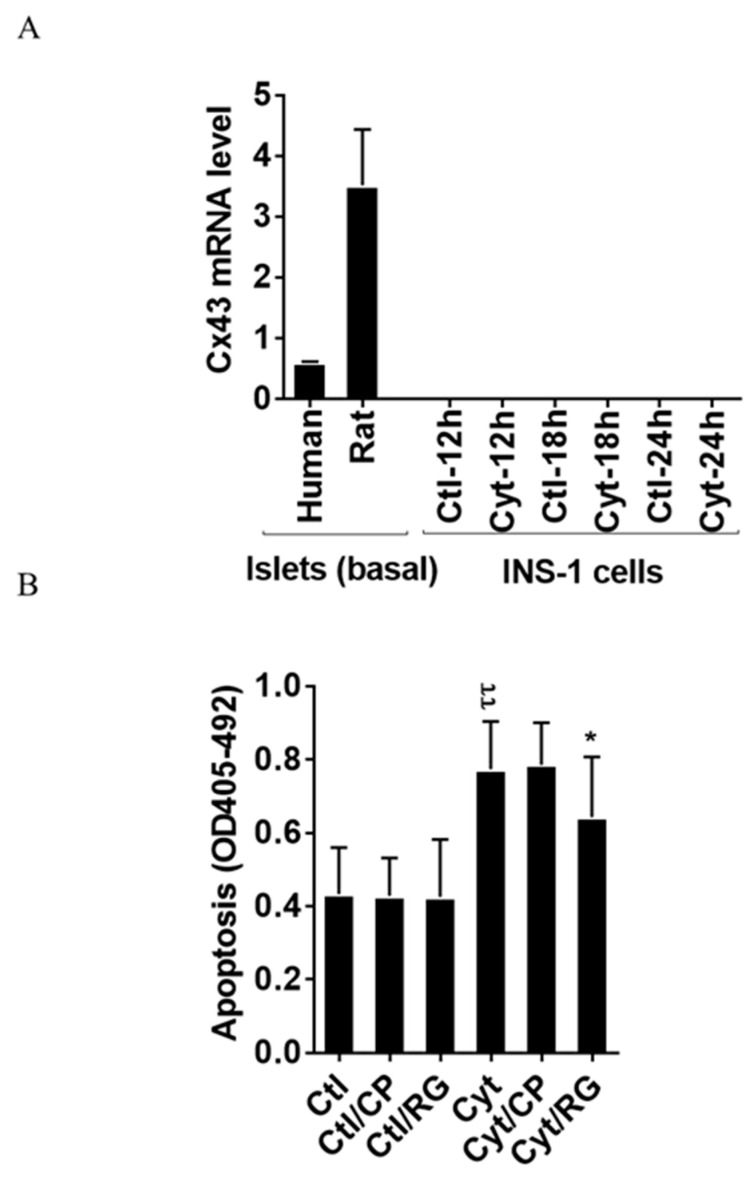
Rotigaptide reduces cytokine-induced apoptosis in Cx43-expressing human islets. Fifty pancreatic islets per condition were pre-incubated with or without 500 nM rotigaptide/ZP123 (RG) or control peptide ZP119 (CP) for one hour in the presence or absence of cytokine mixture (300 pg/mL IL-1β + 10 ng/mL IFNɣ + 10 ng/mL TNFα; Cyt) for 4 days. (**A**) The Cx43 expression level was determined in untreated human and rat islets, and in INS-1 cells exposed to the cytokine combination (150 pg/mL IL-1β +0.1 ng/mL IFNɣ) using specific primers with qPCR. The expression of the genes normalized to HPRT1 was calculated by −∆Ct. (**B**) Apoptosis was measured by Roche cell death assay according to the manufacturer’s protocol. Data are means ± SEM of *n* = 5 donor human islets. * ≤ 0.05, ττ ≤ 0.01. The symbols τ and * indicate the Bonferroni-corrected paired *t*-test values of cytokine (Cyt) exposure versus control (CTL) and Cyt exposure versus exposure to Cyt and RG, respectively. NS: not significant.

As shown in Figure 1A, we found that human and rat islets did express Cx43, whereas INS-1 cells did not, neither in the unchallenged not the cytokine-challenged state. Reanalyzing published human islet single cell sequencing data [32], the expression of connexins apart from Cx36, well-known to be expressed in islet endocrine cells, was determined (Figure S1). Endocrine alpha, beta, and delta cells equally expressed Cx31.9, and 32; acinar and ductal cells equally expressed Cx26, 31, 32, and 43; ductal cells also expressed Cx30.3 and Cx45; pancreatic stellate cells expressed Cx31.9, 43, and 45; whereas endothelial cells were only found to express Cx43 (with the caveat that the number of passenger endothelial cells in cultured human islets is low). We next examined whether the Cx43 activator RG protects human islets from inflammatory-induced cell death. As expected, the cytokine combination increased islet apoptosis by two-fold after 4 days of exposure (Figure 1B). Neither RG nor CP in the absence of cytokines caused apoptosis in human islets. Interestingly, RG significantly reduced cytokine-induced islet apoptosis by 40%, whereas the control peptide did not significantly change cytokine-induced apoptosis.

### 2.2. Rotigaptide Ameliorates Cytokine-Induced Apoptosis Associated with Improved Mitochondrial Function in Cx43-Deficient INS-1 Cells

**Figure 2:**
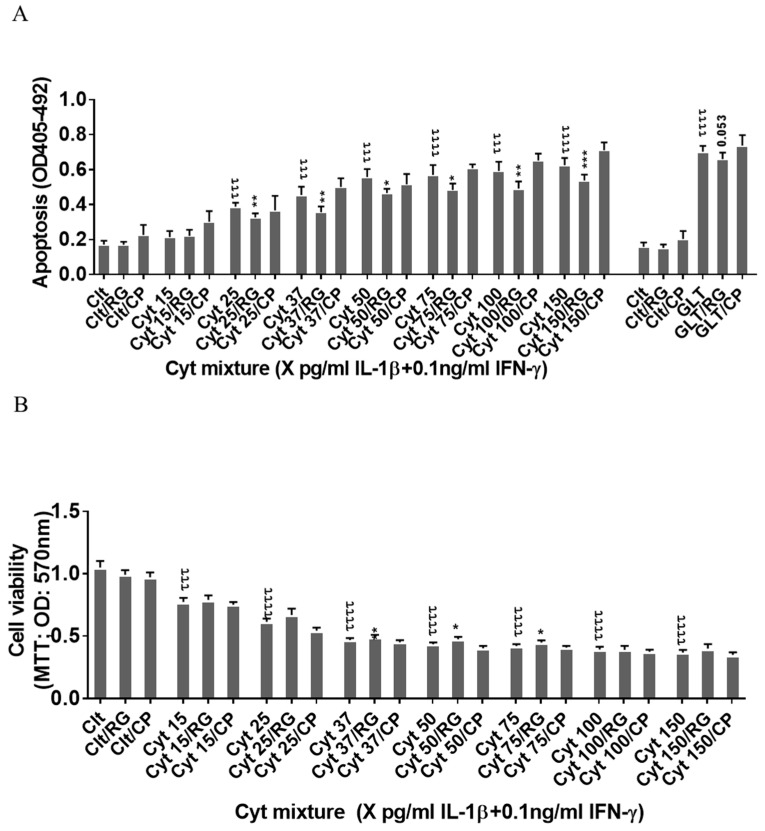
Rotigaptide ameliorates inflammation-induced apoptosis associated with improved mitochondrial function in Cx43-deficient INS-1 cells. INS-1 cells were pre-incubated with or without 100 nM rotigaptide (RG) or control peptide ZP119 (CP) for one hour in the presence or absence of cytokine combination (IL-1β in the concentrations indicated + 0.1 ng/mL IFNγ; Cyt) or glucolipotoxicity (0.5 µM palmitate + 25 mM glucose; GLT) for 24 h. (**A**) Apoptosis was measured by Roche cell death assay according to the manufacturer’s protocol. (**B**) Mitochondrial function was determined using MTT assay. Results are means ± SEM of *n* = 6 independent experiments. * or τ ≤ 0.05, ** or ττ ≤ 0.01, *** or τττ ≤ 0.001, ττττ ≤ 0.0001. The symbols τ and * star indicate the Bonferroni-corrected paired *t*-test values of cytokine (Cyt) exposure versus control (CTL) and Cyt exposure versus exposure to Cyt and RGs, respectively. NS: not significant.

Since the rat insulin-producing INS-1 cells did not express Cx43, and Cx43 was not induced by cytokine exposure for 12–24 h (Figure 1A), INS-1 cells were used a natural Cx43 loss-of-function cell model to investigate Cx43-independent effects of rotigaptide on insulin-producing cells. IL-1β concentrations above 15 pg/mL combined with a fixed concentration of 0.1 ng/mL IFNɣ dose-dependently induced apoptosis in INS-1 cells, with a peak three-fold induction at 150 pg/mL IL-1β (Figure 2A). RG or CP did not by themselves affect INS-1 cell apoptosis. We anticipated that rotigaptide would not protect Cx43-deficient insulin-producing INS-1 cells against cytokine-induced apoptosis to the same extent as that observed in human islets. Unexpectedly, RG but not CP modestly but significantly reduced apoptosis in cytokine-exposed cells by ~10% at IL-1β concentrations above 15 pg/mL. Exposure of INS-1 cells to glucolipotoxic conditions significantly increased apoptosis by 3.8-fold, but this was counteracted neither by RG nor CP.

Since cytokines induce mitochondrial stress in pancreatic beta cells, we then investigated if the Cx43-independent action of RG was associated with the prevention of cytokine-induced mitochondrial dysfunction. IL-1β dose-dependently reduced mitochondrial function by 64% at 150 pg/mL IL-1β. RG, but not CP, slightly but significantly improved mitochondrial function in cytokine-exposed cells at 37, 50, and 75 pg/mL IL-1 (Figure 2B).

### 2.3. Rotigaptide Reduces Neither Cytokine nor Glucolipotoxicity-Induced ROS Production in INS-1 Cells

**Figure 3:**
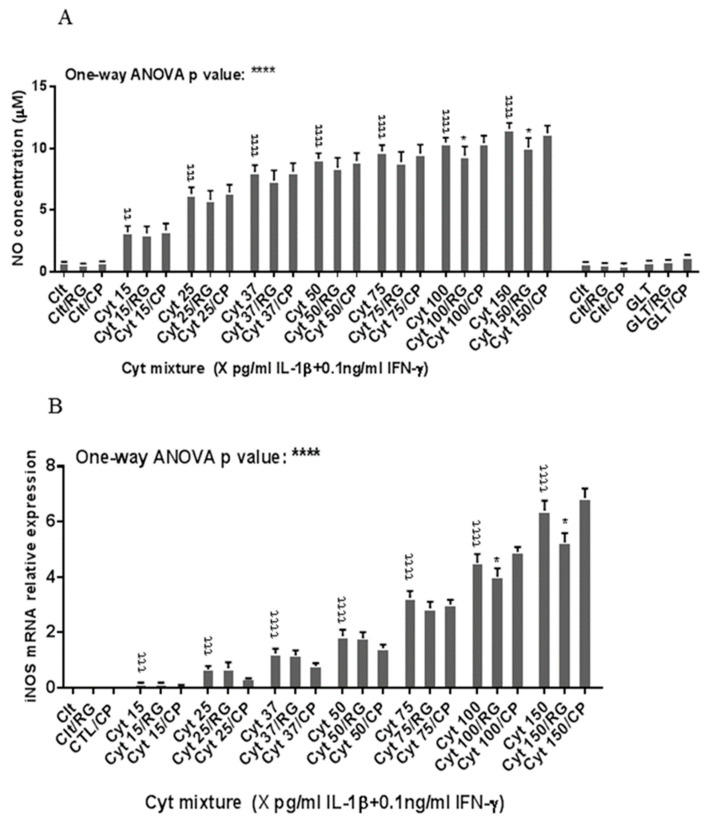
Rotigaptide does not reduce inflammatory or glucolipotoxicity-induced intracellular ROS in INS-1 cells. INS-1 cells were pre-incubated with or without 100 nM rotigaptide (RG) or control peptide ZP119 (CP) for one hour in the presence or absence of cytokine mixture (IL-1β in the concentrations indicated + 0.1 ng/mL IFNɣ; Cyt) or glucolipotoxicity (0.5 µM Palmitate + 25 mM glucose; GLT) for 18 h. (**A**) Cellular ROS level was determined using DCF assay and presented as MFI. (**B**) The mRNA level of NADH-dehydrogenase subunit 2 (*ND2*) and cytochrome C oxidase II (*Sco2)* genes was determined using specific primers with qPCR. The expression of the genes normalized to HPRT1 was calculated by -∆Ct. Data are the means ± SEM of *n* = 6 (for **A**)/*n* = 4 (for **B**) independent experiments. τ ≤ 0.05, ττ ≤ 0.01, τττ ≤ 0.001, ττττ or **** ≤ 0.0001. The symbols τ and * indicate the Bonferroni-corrected paired t-test values of cytokine (Cyt) exposure versus control (CTL) and Cyt exposure versus exposure to Cyt and RG, respectively. NS: not significant. ROS: reactive oxygen species, MFI: mean fluorescent intensity, DCF: dichlorodihydrofluorescein.

We next asked if the improved mitochondrial function caused by RG was associated with reduced ROS production. IL-1β dose-dependently increased INS-1 cell ROS production (Figure 3A), which was unaffected by RG. Additionally, ROS production in response to glucolipotoxic conditions was unaffected by RG. Since oxidative/nitroxidative stress induced by proinflammatory mediators causes dysfunction of mitochondrial complexes by inhibiting their transcription [33], we explored if RG would prevent cytokine-induced mitochondrial dysfunction. We therefore measured the mRNA levels of two genes encoding key subunits of the mitochondrial complex I and IV: NADH-dehydrogenase subunit 2 (*ND2*) and cytochrome C oxidase II (*Sco2*), respectively. Cytokines downregulated ND2, and there was a trend for downregulation of Sco2 (*p* = 0.09), but RG did not restore these changes (Figure 3B).

### 2.4. Rotigaptide Reduces Nitroxidative Stress Independently of Cx43

**Figure 4:**
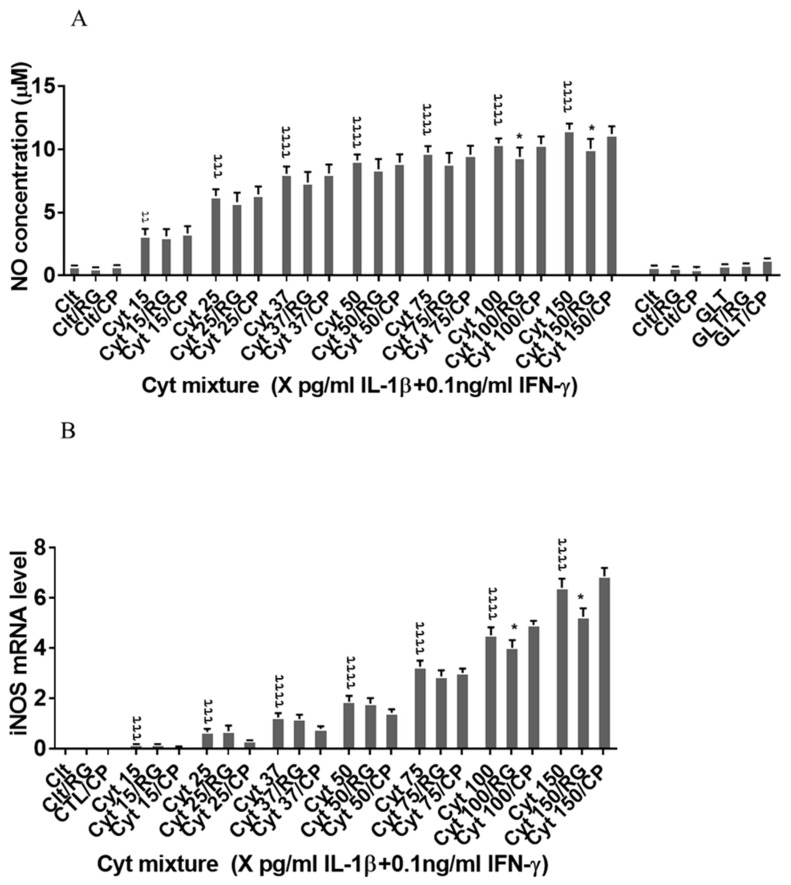

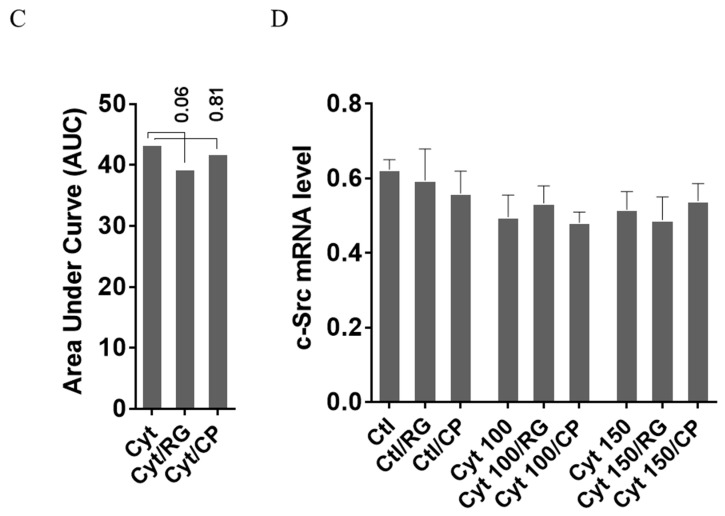
Rotigaptide reduces nitroxidative stress in INS-1 cells. INS-1 cells were pre-incubated with or without 100 nM rotigaptide (RG) or control peptide ZP119 (CP) for one hour in the presence or absence of cytokine mixture for 24 h (**A**), 6 h (**B**,**D**), or in a time course of 5, 10, 15, 20, 25, 30, or 45 min (**C**). (**A**) Accumulated nitrite was measured with Griess reagent in the supernatant. (**B**,**D**) iNOS and c-Src mRNA levels were determined by qPCR. The expression of iNOS and c-Src normalized to HPRT1 was calculated with −∆Ct. (**C**) Immunoblot analysis of the time course of cytokine-induced IkBα degradation in the presence or absence of RG or CP was quantified with ImageJ software and normalized to tubulin. Data are means ± SEM of *n* = 6 (for **A**,**B**,**D**)/*n* = 3 (for **C**) independent experiments. * ≤ 0.05, ττ ≤ 0.01, τττ ≤ 0.001, ττττ ≤ 0.0001. The symbols τ and * indicate the Bonferroni-corrected paired t-test values of cytokine (Cyt) exposure versus control (CTL) and Cyt exposure versus exposure to Cyt and RG, respectively. NS: not significant. AUC: area under curve, NO: nitric oxide, iNOS: inducible nitric oxide synthase, HPRT1: Hypoxanthine-guanine phosphoribosyltransferase 1.

Next, we asked if RG inhibited nitroxidative stress in INS-1 cells. IL-1β significantly induced NO production in INS-1 cells (Figure 4A), which was reduced by RG but not CP at 100 and 150 pg/mL IL-1. IL-1β dose-dependently increased iNOS mRNA levels, which were reduced by RG but not CP at 100 and 150 pg/mL IL-1 (Figure 4B).

We next investigated if rotigaptide mitigated nitroxidative stress by impeding IkBα degradation. Cytokines caused IkBα degradation in a time-dependent manner (Figure 4C). There was a strong trend (*p* = 0.06) for RG but not CP diminishing the AUC for IkBα degradation. Since cytokine-induced c-Src levels might be higher in Cx43-deficient cells and since c-Src binds and thereby acts as a sink for IkBα [34,35], we investigated c-Src expression in INS-1 cells exposed to cytokines in the presence or absence of RG or CP. As shown in Figure 4D, c-Src mRNA levels were unaffected by cytokines and RG.

Taken together, the data suggest that rotigaptide reduces nitroxidative stress independently of its action on Cx43 activity.

### 2.5. Rotigaptide Ameliorates High-Concentration Cytokine-Induced Inhibition of Insulin Secretion and Reduction of Insulin mRNA in INS-1 Cells

**Figure 5:**
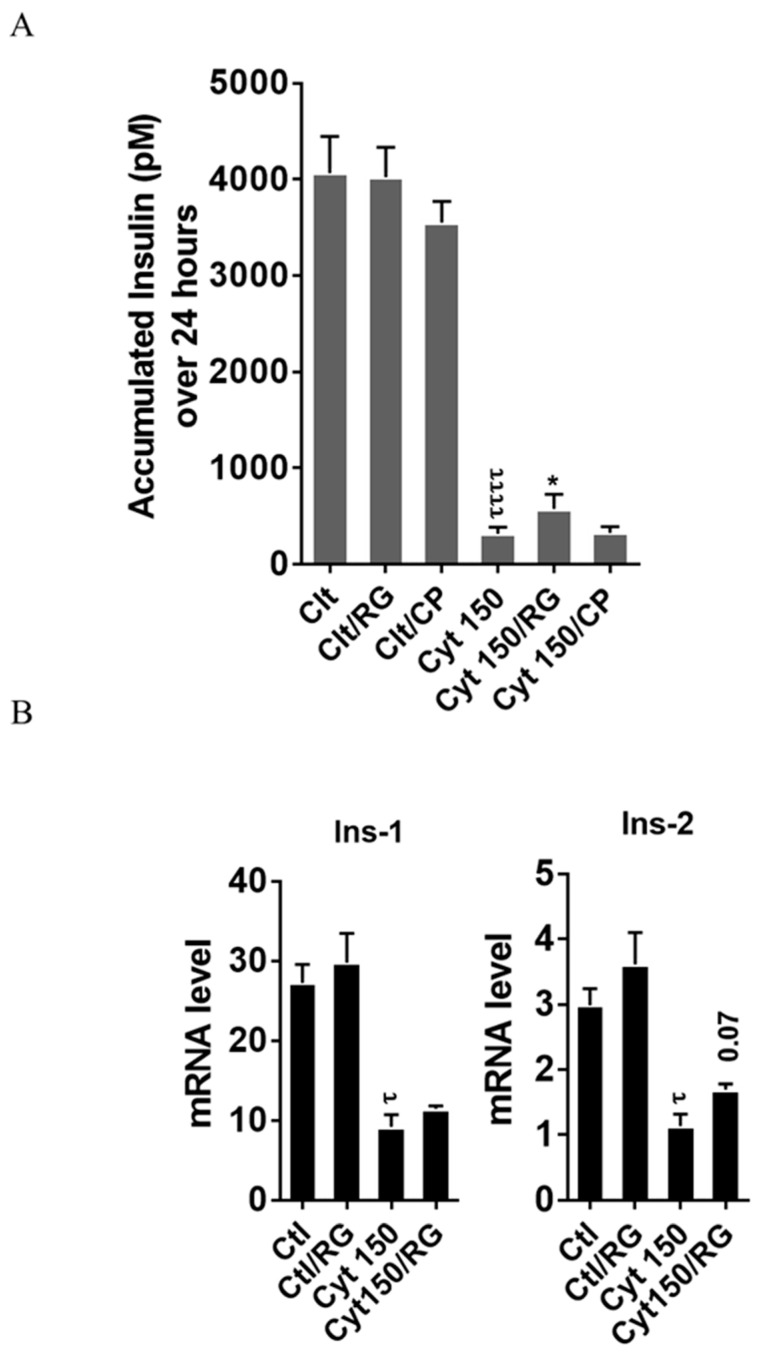
Rotigaptide ameliorates cytokine-induced inhibition of insulin secretion and reduction of insulin mRNA in INS-1 cells. INS-1 cells were pre-incubated with or without 100 nM rotigaptide (RG) or control peptide ZP119 (CP) for one hour in the presence or absence of cytokine mixture for 24 (**A**) or 18 h (**B**). (**A**) Accumulated insulin was measured by competitive insulin ELISA in the supernatant collected. (**B**) The mRNA level of *insulin-1* (Ins-1) and *insulin-2* (Ins-2) genes was determined by qPCR. The expression of the genes normalized to HPRT1 was calculated by −∆Ct. Data are the means ± SEM of *n* = 6 (for A)/*n* = 4 (for B) independent experiments. * or τ ≤ 0.05, ττττ ≤ 0.0001. The symbols τ and * indicate the Bonferroni-corrected paired t-test values of cytokine (Cyt) exposure versus control (CTL) and Cyt exposure versus exposure to Cyt and RG, respectively. NS: not significant.

To explore if RG restores cytokine-mediated inhibition of insulin biosynthesis and secretion, we measured the accumulated insulin secretion and insulin mRNA in INS-1 cells. RG but not CP significantly improved insulin secretion in INS-1 cells at 150 pg/mL IL-1β (Figure 5A).

Finally, we studied if this partial recovery of secreted insulin was due to an increase in the insulin mRNA level. qPCR analysis confirmed significant cytokine-mediated reductions in the mRNA levels of *insulin-1* and *insulin-2*. A trend (*p* = 0.07) for partial reversal of the inhibition in *insulin-2* but not *insulin-1* levels was observed after RG treatment (Figure 5B).

## 3. Discussion

We show here that the Cx43 activator RG reduces proinflammatory cytokine-induced apoptosis in human islets, shown to have low levels of Cx43 expression. Surprisingly, this protective effect was not abrogated in INS-1 cells, shown to be Cx43 deficient (Figure 1A) [20,21]. In these cells, RG still modestly but significantly reduced apoptosis, and also improved mitochondrial function, insulin-2 gene levels, and accumulated insulin release. RG reduced NO production in Cx43-deficient INS-1 cells associated with reduced iNOS expression and IkBα degradation, suggesting that RG blunts cytokine-induced NF-κB signaling in insulin-producing cells in a Cx43-independent manner. High glucose downregulates Cx43 and inhibits the protein–protein interaction between c-Src and Cx43 in glomerular mesangial cells, thereby promoting binding of c-Src to IkBα, in turn activating the NF-κB pathway [34,35]. These observations raise the intriguing possibility that RG may inhibit NF-κB signaling by enhancing Cx43 expression and activity, but this remains to be shown.

An important question is how RG intercepts NF-κB activation in the absence of Cx43. In Cx43-deficient cells, a lack of Cx43/c-Src interaction would be expected to lead to higher c-Src availability for IkBα binding, thereby priming the cells for cytokine-triggered NF-κB activation. We investigated c-Src expression in INS-1 cells and were unable to show altered c-Src mRNA levels caused by either cytokines or RG. In Cx43-competent cells, however, this mechanism would be expected only to play a role under conditions of inhibited Cx43 expression, such as those induced by high glucose [34,35]. The lack of protection of RG on high glucose and high lipid toxicity in INS-1 cells may be explained by the absence of Cx43, and these experiments should therefore be repeated in human islets, in which RG would be expected to counteract a glucose-mediated reduction in Cx43 expression.

Given the lack of beta cell Cx43 expression shown by others [17,18,19,22] and in our single-cell RNA-seq analysis of human islets (Figure S1), but that heterotypic channels can be formed between Cx43-positive non-endocrine pancreatic cells and Cx43-negative islet endocrine cells expressing other connexins, such as Cx36 or Cx32, we suggest that RG promotes the coupling of Cx43 expressed on islet endothelial or pancreatic stellate cells to other islet endocrine cell connexins to form heterotypic channels that promote islet cell survival.

The strengths of this study were the use of human islets to demonstrate the protective effect of RG against cytokine-induced apoptosis, and the inclusion of a scrambled peptide as a control for RG. We also took advantage of INS-1 cells shown to lack Cx43 expression as a ‘natural’ Cx43 knock-out model (Figure 1A) [20,21], thereby avoiding the risk for transfection artifacts related to small hairpin (sh) RNA knock-down or CRISPR knock-out.

A limitation was the restricted amounts of human islets available to us for these studies, precluding more mechanistic investigations, such as Cx43 protein expression and cellular localization, Cx43 channel activity, Ca^2+^ flux synchronization, and studies of glucose-stimulated insulin secretion. Electromobility shift assay, iNOS promotor chromatin immunoprecipitation, or luciferase-based NF-κB reporter assay would help define if NF-κB promoter binding or NF-κB transcriptional activity is reduced in rotigaptide-treated islets exposed to cytokines.

Rotigaptide is known to increase the Cx43 half-life in cardiomyocytes by slowing its trafficking and proteasomal degradation [29,36,37]. However, proof of Cx43 dependence of such effects of RG in human islets would require knock-down or knock-out of Cx43, which is not effective in intact islets but requires islet dispersion into monolayers, thereby disrupting the normal cell-to-cell contact and communication. This study does not demonstrate that RG-mediated prevention of cytokine-induced apoptosis is beta cell specific. Such studies would be demanding, since the sorting of islet cells and even reassembly into beta cell-enriched pseudo-islets would eliminate interaction with Cx43-expressing non-endocrine cells. However, since cytokine-mediated islet cell apoptosis is selective for beta cells [1,38] it is likely that the reduction of human islet cell apoptosis by RG is related to reduced beta cell death. Further research is needed to clarify if these effects contribute to the protective action of rotigaptide in human islets.

When added to the fact that Cx43 is required for beta cell differentiation and maturation [19], our observations highlight the translational potential of RG as a novel approach to prevent inflammatory islet cell failure and apoptosis in diabetes and warrant further preclinical studies. The notion that RG may have a dual protective action related to its known activity as a Cx43 activator and to a novel Cx43-independent inhibiting action on NF-κB activation makes RG an attractive candidate for mono- or combination therapy in diabetes.

## 4. Methods

### 4.1. Reagents

Recombinant rat (rr) IL-1β, mouse (rm) IFNɣ, human (rh) IFNɣ, and rhTNFα were purchased from R&D Systems (Minneapolis, Macheray-Nagel, USA). Rotigaptide (RG; ZP123) and control scrambled inert hexapeptide (CP; ZP119) were provided by Zealand Pharma (Soeborg, Denmark).

### 4.2. Cell Culture and Exposures

The rat INS-1 cell line (generously provided by Claes Wollheim, University of Geneva, Geneva, Switzerland) known not to express Cx43 [20,21] was tested negative for *Mycoplasma* and maintained as previously described [38,39]. INS-1 cells were seeded in 6-well plates (1 × 10^6^ cells/well for RNA isolation), 48-well plates (50,000 cells/well for cell death assay in duplicate), or 96-well plates (30,000 cells/well for MTT assay and ROS assay) (all plates from NUNC, Roskilde, Denmark). After 48 h of pre-incubation, cells were treated for the time periods indicated with or without 100 nM rotigaptide or CP, for one hour and then cultured with or without cytokine mixture (Cyt) at the concentrations indicated in the figures or figure legends, or in glucolipotoxic conditions (0.5 µM palmitate conjugated with 0.1% albumin as described in [40] + 25 mM glucose; GLT) for 24 h. Two concentrations of 100 and 500 nM rotigaptide were tested in INS-1 cells. No differences in the efficacy on cell death and NO were noted, and therefore 100 nM was selected, in agreement with [29].

### 4.3. Human Islet Culture and Exposures

Islets from five human heart-beating organ donors (>80 purity, donor characteristics listed in Table 1) were isolated by the European Consortium for Islet Transplantation (ECIT) in Milan, Italy under local approval and received in fully anonymous form, were cultured in 10% fetal bovine serum (FBS), 1% penicillin/streptomycin (P/S), and 5.6 mM glucose at 5% CO_2_ and 37 °C as described previously [41]. There were no apparent differences in the results obtained with islets from male or female donors, and data were therefore combined.

Fifty human islets were pre-cultured in RPMI supplemented with 2% human serum (Life Technologies, Naerum, Denmark), 1% penicillin/streptomycin (P/S), and 5.6 mM glucose for 24 h, treated with or without 500 nM rotigaptide or control peptide for one hour and then exposed to cytokine mixture (300 pg/mL rrIL-1β +10 ng/mL rhIFNɣ + 10 ng/mL rhTNFα) or control medium for 4 days.

### 4.4. Apoptosis and Cell Viability Assays

For the apoptosis assay carried out in duplicate independent cultures, DNA/histone complexes released from the nucleus to the cytosol were measured using a Roche cell death assay kit (Roche, Mannheim, Germany) according to the manufacturer’s protocol. As a surrogate of cell viability, mitochondrial function was measured in duplicate by the MTT assay in which water-soluble *MTT* (3-(4,5-dimethylthiazol-2-yl)-2,5-diphenyltetrazolium-bromide) was converted by normal cells to an insoluble formazan salt with an optical density (OD) read at 570 nm.

### 4.5. Nitric Oxide (NO) Assay

As a surrogate of nitric oxide production, accumulated nitrite was measured in duplicate samples from two independent parallel cultures. Supernatants (100 µL) from the wells used for the INS-1 cell apoptosis assay were mixed with an equal volume of the Griess reagent (one part 0.1% naphtylethylene diamine dihydrochloride and one part 1% sulfanilamide in 5% H3PO4 (Merck, Darmstadt, Germany), and read at 550 nm in a plate reader (Thermo Scientific, Naerum, Denmark). The nitrite concentration was calculated using a standard curve of 0.5–40 µM concentrations of NaNO_2_ (Merck, Darmstadt, Germany) as described [42].

### 4.6. Reactive Oxygen Species (ROS) Assay

ROS was measured in triplicate independent parallel cultures by the 5-(and-6)-chloromethyl-20,70-dichlorodihydrofluorescein (H2DCFDA) (Invitrogen, Naerum, Denmark) probe as previously described [43]. Fluorescence was read at excitation 495 nm/emission 527 nm and results shown as the delta fluorescence for the time interval of 90–45 min.

### 4.7. Real-Time Quantitative RT-PCR

Total RNA was extracted using the Nucleo-Spin kit (Macheray-Nagel, Bethlehem, USA) according to the manufacturer’s instructions. The quality and quantity of the extracted RNA was assessed using a NanoDrop-1000 (Thermo Scientific). In total, 500 ng of total RNA was used for cDNA synthesis with the iScript™-cDNA Kit (BioRad, Copenhagen, Denmark). Real-time qPCR was performed on 12 ng cDNA in triplicate with SybrGreen PCR mastermix (Life Technologies, Naerum, Denmark) and specific primers (Table 2) and run in a Real-Time PCR machine (Applied Biosystems, Naerum, Denmark). The gene expression level was normalized to HPRT1 through −∆Ct analysis [44].

### 4.8. Western Blot Analysis

Cells were lysed on ice with NP-40 lysis buffer containing protease inhibitor cocktail (Life Technologies) and stored in a −20 freezer. Lysates were adjusted for protein concentration with the Bradford assay according to the manufacturer’s protocol (BioRad, Copenhagen, Denmark), and 50 µg of protein separated by 4–20% SDS-PAGE, and blotted on PDVF membrane (BioRad, Copenhagen, Denmark). The membranes were stained with anti-IkB (Santa Cruz, Heidelberg, Germany) and alpha-tubulin (Sigma, Copenhagen, Denmark) antibodies and developed with the chemiluminescence detection system Super Signal (Life Technologies, Naerum, Denmark) as previously described [45]. Light emission was captured using an Alphaimager system (Alpha Innotech, MultiImage III, Broager, Denmark). Band density was quantified using ImageJ software.

### 4.9. Insulin Assay

Supernatants (1:200 dilution) collected from the INS-1 cell apoptosis assay experiments were used for the measurement of accumulated insulin using the insulin competitive ELISA assay in duplicate samples from two independent parallel cultures as described [46], except that the enzyme substrate 1-step Ultra TMB (3,3′, 5,5;-tetramethylbenzidine) (Life Technologies, Naerum, Denmark) was used here.

### 4.10. Single-Cell RNA-Seq of Human Pancreatic Islets

The expression of genes in islet cell types was determined by reanalyzing published human islet single-cell sequencing data (donor information in EBI accession number: MTAB-5061) [32] as previously described [47].

### 4.11. Statistical Analysis

Data are presented as means ± SEM, and comparisons between different groups were carried out by ANOVA analysis, followed by Student’s paired *t* test using the GraphPad Prism version 6 (La Jolla, CA, USA). Bonferroni-corrected *p*-values ≤ 0.05 were considered as significant and ≤0.10 as a trend.

## 5. Conclusions

RG reduces cytokine-induced cell death in human islets, likely by preventing Cx43 uncoupling. RG conferred protection against inflammatory assault even in Cx43-deficient INS-1 cells, suggesting a novel inhibitory mechanism of action of RG on NF-κB signaling. These observations support further development of RG as a novel therapy to protect the islet functional mass in diabetes due to its dual protective action on key islet cell pro-apoptotic pathways.

## Figures and Tables

**Table 1:** Characteristics of human donors of pancreatic islets.

No.	Lab Code	Gender	Age	BMI
1	HE-5-15	F	22	22.8
2	HE-10-16	F	60	23.6
3	HE-15-16	M	34	23.1
4	HE-17-16	M	50	24.8
5	HE-2-17	M	52	25.1

**Table 2:** Rat primer sequences.

**Rat Primers**		
Target	Forward	Reverse
iNOS	CACCACCCTCCTTGTTCAACA	CAATCCACAACTCGCTCCAA
Ins-1	TCTGCTCCCTCTACCAACTG	TGCTCATTCAAAGGCTTTATTCAT
Ins-2	CCCTAAGTGACCAGCTACAG	GAAGCGGATCCACAGGGC
HPRT1	GCAGACTTTGCTTTCCTT	CCGCTGTCTTTTAGGCTT
Sco2	TGGCTTACCCATTTCAACTTGG	GATGAGGAATACAATTATTAGGGTGTG
ND2	AAACCCAATCACCCTAATCATTA	CAATCCTACTCATATTAGGAGTAAGT
c-Src	GGCCCAAGTCATGAAGAAAC	CATGCCTGAAGCAATCTGAG
Cx43	CGCAATTACAACAAGCAAGC	TCATGTCCAGCAGCAACTTT
**Human Primers**		
Target	Forward	Reverse
Cx43	ATGAGCAGTCTGCCTTTCGT	TCTGCTTCAAGTGCATGTCC
HPRT1	ATG CTG AGG ATT TGG AAA GG	TAA TCC AGC AGG TCA GCA AA

## Supplementary Materials

The following are available online at https://www.mdpi.com/1422-0067/21/12/4311/s1.
