# Supplementary material for: The Connexin 43 Regulator Rotigaptide Reduces Cytokine-Induced Cell Death in Human Islets
Source: Int J Mol Sci. 2020 Jun 17;21(12):4311. doi: 10.3390/ijms21124311 (PMC7352593; doi:10.3390/ijms21124311)
Supplement: Supplementary file 1 [file ijms-21-04311-s001.pdf]

International Journal of
Molecular Sciences

MDPI

Supplementary Data

Article

The Connexin 43 Regulator Rotigaptide Reduces Cytokine-Induced Cell Death in Human Islets

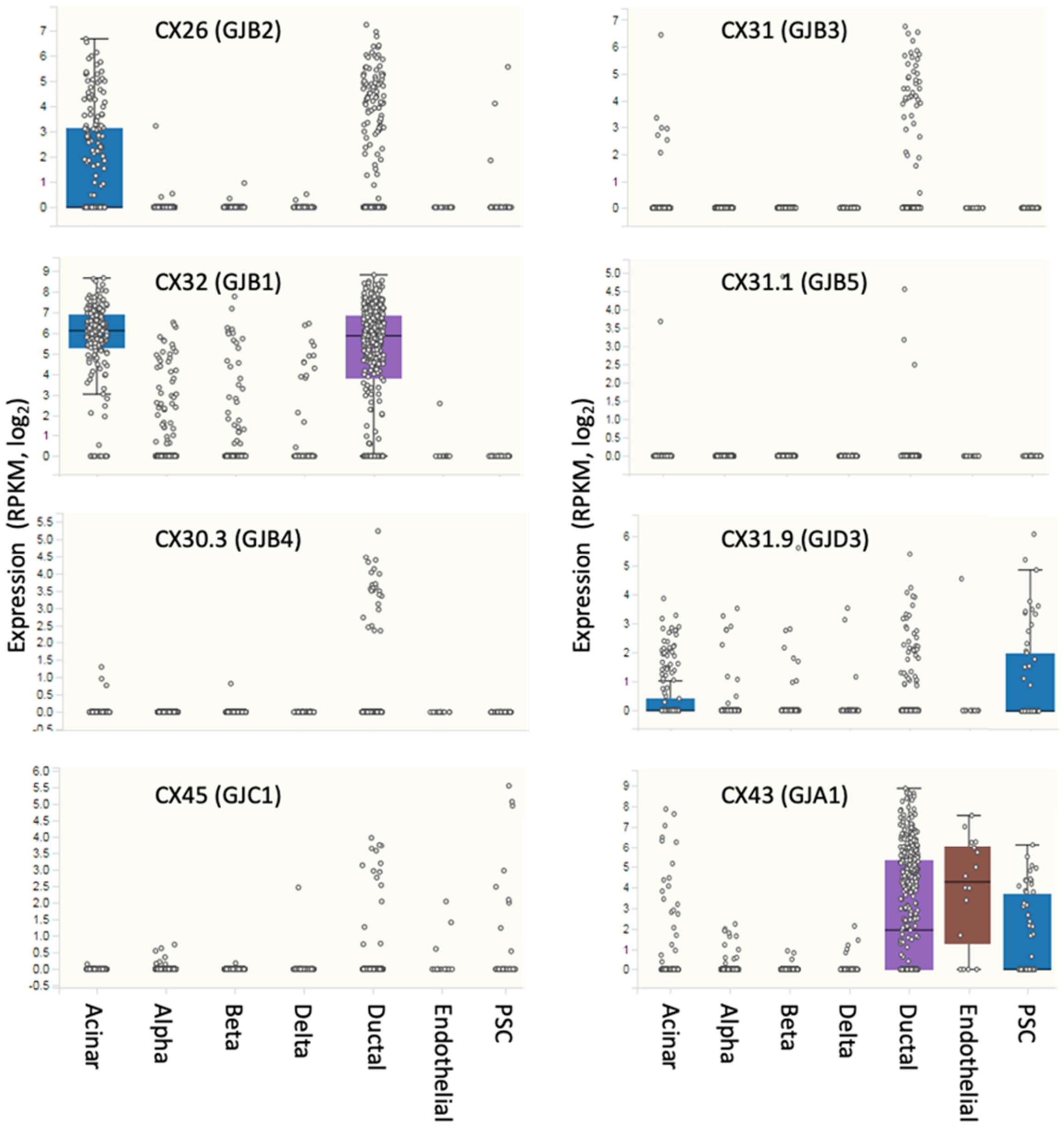

Figure S1. Single cell RNA sequencing of dispersed human islets from six healthy donors. Each transcript is plotted per cell. All donors are merged together. The total number of cells per group can be found in the original publication [32]. The HGNC gene symbol is given in parenthesis. Abbreviation: Cx: connexin, PSC: pancreatic stellate cells.
